# FT3/FT4 ratio is correlated with all-cause mortality, cardiovascular mortality, and cardiovascular disease risk: NHANES 2007-2012

**DOI:** 10.3389/fendo.2022.964822

**Published:** 2022-08-18

**Authors:** Xueyan Lang, Yilan Li, Dandan Zhang, Yuheng Zhang, Nilian Wu, Yao Zhang

**Affiliations:** ^1^ Department of Cardiology, the Second Affiliated Hospital of Harbin Medical University, Harbin, China; ^2^ Key Laboratory of Myocardial Ischemia, Ministry of Education, Harbin Medical University, Harbin, China

**Keywords:** NHANES, FT3/FT4 ratio, all-cause mortality, cardiovascular mortality, cardiovascular disease

## Abstract

**Background:**

Thyroid hormones play a vital role in maintaining the homeostasis of the cardiovascular system. The FT3/FT4 ratio can be used to evaluate the rate of T4-to-T3 conversion, reflecting the peripheral sensitivity of thyroid hormones. There is no study to investigate its relationship with death and cardiovascular disease (CVD) in the general population.

**Methods:**

This retrospective cohort study involved 8,018 participants with measured thyroid function and no prior thyroid disease who participated in the National Health and Nutrition Examination Survey (NHANES) from 2007 to 2012. Mortality status was determined by routine follow-up using the National Death Index through December 31, 2015.

**Results:**

During a median of 87 months of follow-up, we observed 699 all-cause deaths, including 116 cardiovascular deaths. In multivariate adjusted models, higher free thyroxine (FT4) was linked to increased all-cause mortality (HR, 1.15 per SD; 95% CI, 1.09-1.22), cardiovascular mortality (HR, 1.18 per SD; 95% CI, 1.01-1.39), and CVD risk (HR, 1.17 per SD; 95% CI, 1.08-1.27). Higher free triiodothyronine (FT3) was linked to decreased all-cause mortality (HR 0.81 per SD; 95% CI, 0.70-0.93). Higher FT3/FT4 ratio was linked to decreased all-cause mortality (HR, 0.77 per SD; 95% CI, 0.69-0.85), cardiovascular mortality (HR, 0.79 per SD; 95% CI, 0.62-1.00), and CVD risk (HR, 0.82 per SD; 95% CI, 0.74-0.92). The FT3/FT4 ratio stratified findings were broadly consistent with the overall results.

**Conclusions:**

FT3, FT4, and the FT3/FT4 ratio were all independent predictors of all-cause death. FT4 and the FT3/FT4 ratio, but not FT3, were independent predictors of cardiovascular mortality and CVD risk. Along with FT3 and FT4, we should pay equal attention to the FT3/FT4 ratio in the general population.

## Introduction

Thyroid hormones are major metabolic regulators that have a complicated connection with the cardiovascular system through a variety of processes, including endothelial function, blood pressure fluctuations, myocardial function, and blood lipid levels (1). Thyroid hormones in the circulation mainly exist in two states: triiodothyronine (T3) and thyroxine (T4). T3 is mainly converted from T4 by deiodinase iodothyronine in peripheral tissues (2). There is increasing evidence that thyroid dysfunction is linked to poor prognosis in patients with heart disease (3–7). Even in subjects without obvious thyroid disease, too much or too little thyroid hormone can accelerate the progression of cardiovascular disease and even accelerate death. For example, in one observational study, high free thyroxine (FT4) and low free triiodothyronine (FT3) levels had a strong correlation with all-cause and cardiovascular mortality in patients without overt thyroid disease and receiving coronary angiography (8). Ataoglu HE et al. showed that among hospitalized chronic patients with nonthyroid illness syndromes, both low FT3 and high FT4 levels were independent indicators of mortality risk (9). However, in the general population, the impacts of FT3 and FT4 on the prognosis of subjects without overt thyroid disease were inconsistent.

There are three main iodothyronine deiodinases in the body: iodothyronine deiodinase 1 (DIO1) and iodothyronine deiodinase 2 (DIO2) 5′-deiodinases catalyzes the activation reaction from T4 to T3, iodothyronine deiodinase 3 (DIO3) 5-deiodinase catalyzes the activation reaction from T4 inactivation response to reverse T3 (10). FT3/FT4 reaction 5’-deiodinase activity can be used to evaluate peripheral thyroid sensitivity (2, 11–14). The FT3/FT4 ratio seems to be a precise and reasonable indicator of thyroid hormone metabolic change that is associated with the prognosis of specific diseases, including coronary heart disease (CHD), myocardial infarction (MI), and dilated cardiomyopathy (DCM) (15–18). However, there are no data on the value of the FT3/FT4 ratio in predicting mortality risk in the general population, especially in those with normal thyroid function.

Cardiovascular disease (CVD) is a group of heart and vascular diseases mainly represented by CHD, angina pectoris, congestive heart failure (CHF), MI and stroke (19). Thyroid hormones are critical for cardiovascular homeostasis (20). Several cross-sectional investigations have shown that in the general population with normal thyroid function, thyroid hormone levels are correlated with coronary artery calcification (21), arterial stiffness (22) and the incidence of metabolic syndrome (23). Cappola AR et al. found that in the general population, higher baseline FT4 was correlated with higher rates of CHD and heart failure during follow-up (24), but the relationship between thyroid hormone levels and CVD in the general population remains unknown.

The purpose of this research was to investigate the probable relationship between the FT3, FT4, or FT3/FT4 ratio and all-cause, cardiovascular mortality, and CVD risk in the general population of the U.S. without previous thyroid illness using a representative national sample.

## Methods

### Study population

The National Health and Nutrition Examination Survey (NHANES) is a survey that uses a nationally representative sample to examine the nutritional and health conditions of the civilian population in the United States. The National Center for Health Statistics Research Ethics Review Board (Hyattsville, MD, USA) authorized the survey procedure, and all participants provided signed informed consent. NHANES provides detailed information over the web (www.cdc.gov/nchs/nhanes/index.htm; viewed on January 12, 2022).

**Figure 1:**
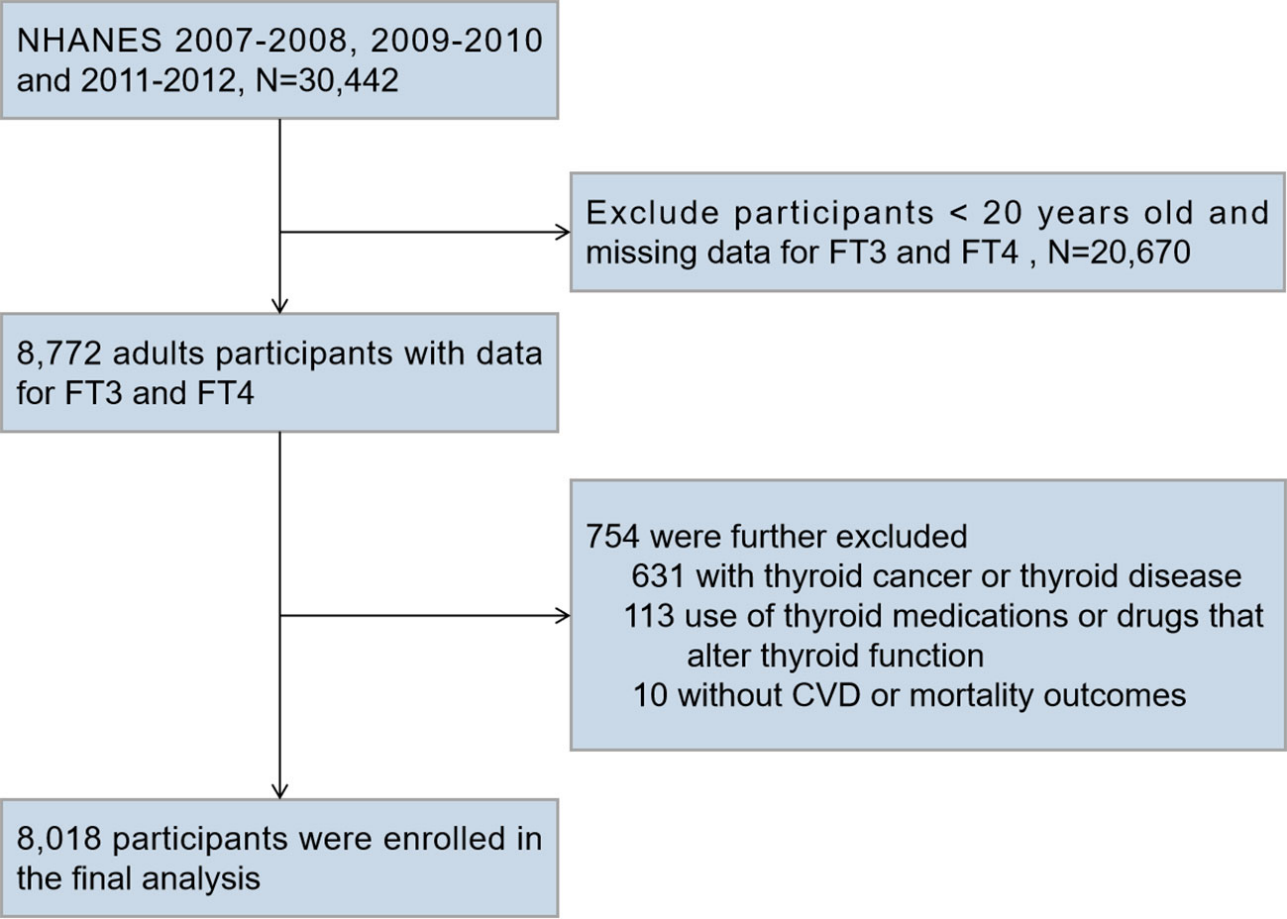
Flow Chart for Subject Selection. NHANES, National Health and Nutrition Examination Survey; FT3, free triiodothyronine; FT4, free thyroxine; CVD, cardiovascular disease.

We merged three cycles of NHANES data from 2007 to 2012 for this research (N = 30,442). We included participants aged 20 years or older who had a serum FT4 and FT3 test (N = 8,772). We excluded participants who reported a history of thyroid cancer or thyroid disease (N = 631), participants taking thyroid drugs (liothyronine, levothyroxine, propylthiouracil, or methimazole) or medications that alter thyroid function tests (amiodarone, corticosteroids, and lithium) (N = 113), and those without CVD or mortality outcome data (N = 10). **Figure 1** depicts the full data integration process. There were a total of 8,018 people in the final analysis.

### Determination of serum thyroid function

Serum FT3 was detected by a competitive binding immunoenzymatic assay, and serum FT4 was detected by a two-step enzyme immunoassay. The reference range for thyroid hormone levels in NHANES 2007-2012 was a serum FT4 level of 0.6-1.6 ng/dL and a serum FT3 level of 2.5-3.9 ng/dL. The monoclonal anti-T4 antibody coupled to biotin, sample, buffered protein solution, and streptavidin-coated solid phase are added to the reaction vessel. During this first incubation, the anti-T4 antibody coupled to biotin binds to the solid phase and the FT4 in the sample. After incubation in a reaction vessel, materials bound to the solid phase are held in a magnetic field while unbound materials are washed away. Next, buffered protein solution and T3-alkaline phosphatase conjugate were added to the reaction vessel. The T3-alkaline phosphatase conjugate binds to the vacant anti-T4 antibody binding sites. After incubation in a reaction vessel, materials bound to the solid phase are held in a magnetic field while unbound materials are washed away. Further detailed quality control steps can be found on the NHANES website in the Mobile Examination Center (MEC) laboratory procedure manual. FT4 is converted in peripheral tissues to active FT3, which may be measured by the FT3/FT4 ratio (25).

### Ascertainment of outcomes

National Center for Health Statistics mortality was determined using a probabilistic record match between NHANES participants and National Death Index (NDI) death certificate data. Through December 31, 2015, the NHANES-linked NDI public access data was used to identify the mortality status and cause of death.

CVD was diagnosed using self-reported physician diagnoses obtained during an individual interview using a standardized medical condition questionnaire. The participants were asked, “Has a doctor or other health expert ever informed you that you have CHF/CHD/angina pectoris/MI/stroke?” A person was regarded as having CVD if he or she replied “yes” to any of the above questions. The result was transformed to a binary variable.

### Assessment of covariates

The following demographic and lifestyle variables were obtained by standardized questionnaires during home interviews: age, sex, race/ethnicity, educational level, and marital status. Consumption of alcoholic beverages, body weight, and height were determined during physical exams conducted at the MEC. Age was categorized into 6 groups (ages 18-29, 30-39, 40-49, 50-59, 60-69, and ≥ 70 years). Body mass index (BMI) was calculated by dividing the weight in kilograms by the square of the height in meters, and subjects were classified into 3 groups (< 25, 25-29.9 and ≥ 30 kg/m^2^). Race/ethnicity was categorized as Mexican American, non-Hispanic white, non-Hispanic black, and other. Marital status was categorized as married/living with partner, divorced/widowed/separated, and never married. Educational attainment was classified as less than high school, high school graduate/general education development (GED), and a college degree or more. Serum cotinine was applied to measure exposure to environmental smoking and was categorized as follows: low-exposed nonsmokers (serum cotinine < limit of detection), highly exposed nonsmokers (serum cotinine was between the limit of detection and ≤ 10.0 ng/mL), and exposed smokers (serum cotinine > 10.0 ng/mL). Alcohol consumption was defined as regular drinking in the past 12 months (yes or no).

In addition, the Chronic Kidney Disease Epidemiology Collaboration (CKD-EPI) equation was utilized to compute the estimated glomerular filtration rate (eGFR) (26). Normal renal function was defined as an eGFR ≥ 90 mL/min/1.73 m^2^, and renal impairment was defined as an eGFR < 90 mL/min/1.73 m^2^ (27). Hypertension was defined as a self-reported medical diagnosis of hypertension, self-reported use of blood pressure drugs, ≥ 140 mmHg measured systolic blood pressure or ≥ 90 mmHg measured diastolic blood pressure. Diabetes was defined as a self-reported medical diagnosis of diabetes, the intake of antidiabetic medications or insulin, a glycated hemoglobin (HbA1c) level of ≥ 6.5%, a fasting glucose level of ≥ 7.0 mmol/L, or a two-hour glucose level of ≥ 11.1 mmol/L after an oral glucose tolerance test. Hypercholesterolemia was defined as total cholesterol ≥ 6.21 mmol/L, a documented history of hypercholesterolemia, or the use of lipid-lowering drugs.

### Statistical analysis

According to NHANES analytic standards, all analyses used sample weights to account for the complicated survey design. Continuous variables that were normally or approximately normally distributed are reported as mean (standard deviation, SD) and those with a skewed distribution as median (interquartile range, IQR), while categorical variables are number (percentage, %). With regard to categorical variables, we used the chi-squared test; for normal continuous variables, we used one-way ANOVA; and for skewed continuous variables, we used the Kruskal-Wallis test.

Assessment of nonlinear associations of FT3, FT4, or FT3/FT4 with all-cause and cardiovascular mortality and CVD risk using the restricted cubic spline method (3 nodes, the 25th percentile as a reference point).

The Cox proportional hazards models were employed to estimate the hazard ratios (HRs) and 95% confidence interval (CI) of all‐cause and cardiovascular mortality for each SD increment of the FT3, FT4 or FT3/FT4 ratio. To look into putative links between thyroid hormone levels and CVD and subtypes of CVD (CHF, CHD, angina pectoris, MI, and stroke), we used binary logistic regression to calculate the odds ratios (OR) and 95% CI per SD increase in the FT3, FT4, or FT3/FT4 ratio. Model 1 included adjustments for age and sex. Model 2 added race/ethnicity, education, marital status, serum cotinine, and alcohol drinking. Model 3 was further adjusted for BMI, hypertension, diabetes, hypercholesterolemia, renal impairment, history of CVD, history of cancer, and history of liver impairment, thyroid peroxidase antibodies (TPOAb), thyroglobulin antibodies (TgAb), and thyroid-stimulating hormone (TSH). Model 3 for CVD and CVD subtype risk replaced CVD with a family history of CVD. Schoenfeld residuals were employed to verify the proportional hazards assumption, and no substantial breaches of the assumption were discovered.

Stratified analyses were performed on the FT3/FT4 ratio according to age (< 60 or ≥ 60 years), sex (male or female), BMI (< 25 or ≥ 25 kg/m^2^), hypertension (yes or no), diabetes (yes or no), hypercholesterolemia (yes or no), and renal impairment (yes or no). Multiplicative interactions were introduced to determine whether the correlation between FT3/FT4 ratio and all-cause mortality, cardiovascular mortality, and total CVD varied due to the underlying factors mentioned above.

For the analyses, R version 3.6.1 (R Institute for Statistical Computing, Vienna, Austria), Stata Statistical Software (Version 15.1; Stata Corp, College Station, TX, USA), and IBM SPSS Statistics (Version 25; IBM, Armonk, NY, USA) were used for the analyses. P<0.05 was considered statistically significant.

## Results

### Participant characteristics

The baseline characteristics of participants by quartile of serum FT3/FT4 ratio are presented in **Table 1**. In our study, 8,018 participants participated in the analysis, including 789 participants with CVD. During a median 87-month (IQR 63-97) follow-up, there were 699 all-cause deaths and 116 cardiovascular deaths. The average age of the subjects was 48.93 (17.62) years, and 48.50% were male. The levels of FT3, FT4 or the FT3/FT4 ratio were approximately normally distributed, with an average FT3 level of 3.18 (0.56) ng/dl, FT4 of 0.79 (0.16) ng/dl, and a FT3/FT4 ratio of 4.13 (0.88). Subjects with higher FT3/FT4 ratios were younger, female, never married, obese, had higher cotinine levels, a higher proportion of subjects drank alcohol, had less education, were less likely to be non-Hispanic, and had fewer comorbidities such as cancer, renal impairment, hypertension, diabetes, CVD, and CVD subtypes.

**Table 1:** Baseline characteristics of participants according to the FT3/FT4 ratio in NHANES III (2007-2012).

Characteristic	Total	Quartile 1	Quartile 2	Quartile 3	Quartile 4	*P* value
		(< 3.589)	(3.590-4.064)	(4.065-4.570)	(≥ 4.571)	
Participants, No.	8018	2004	2003	1887	2124	
Age, mean (SD), y	48.93 (17.62)	55.95 (18.21)	49.40 (17.65)	46.06 (16.65)	44.41 (15.63)	< 0.001
Sex (men)	3889 (48.50)	1110 (55.39)	1037 (51.77)	865 (45.84)	877 (41.29)	< 0.001
FT4, mean (SD), ng/dl	0.79 (0.16)	0.93 (0.18)	0.81 (0.08)	0.76 (0.12)	0.67 (0.11)	< 0.001
FT3, mean (SD), ng/dl	3.18 (0.56)	2.95 (0.41)	3.11 (0.31)	3.26 (0.52)	3.41 (0.75)	< 0.001
TSH, mean (SD), µIU/ml	1.55 (1.05-2.31)	1.50 (1.03-2.19)	1.55 (1.05-2.27)	1.57 (1.06-2.31)	1.60 (1.06-2.42)	< 0.001
Race/ethnicity						< 0.001
Mexican American	1332 (16.61)	264 (13.17)	315 (15.73)	336 (17.81)	417 (19.63)	
Non-Hispanic white	1494 (18.63)	426 (21.26)	374 (18.67)	317 (16.80)	377 (17.75)	
Non-Hispanic black	3554 (44.33)	903 (45.06)	878 (43.83)	873 (46.26)	900 (42.37)	
Other	1638 (20.43)	411 (20.51)	436 (21.77)	361 (19.13)	430 (20.25)	
Education						< 0.001
< High school	2351 (29.35)	584 (29.22)	542 (27.07)	568 (30.12)	657 (30.96)
High school/GED	1872 (23.37)	407 (20.36)	455 (22.73)	451 (23.91)	559 (26.34)
> High school	3786 (47.27)	1008 (50.43)	1005 (50.20)	867 (45.97)	906 (42.70)
Marital Status						< 0.001
Married/unmarried couple	4824 (60.18)	1176 (58.71)	1226 (61.21)	1127 (59.76)	1295 (60.97)
Divorced/widowed/separated	1743 (21.74)	530 (26.46)	438 (21.87)	392 (20.79)	383 (18.03)
Never married	1449 (18.08)	297 (14.83)	339 (16.93)	367 (19.46)	446 (21.00)
Alcohol drinking	5333 (72.64)	1224 (67.14)	1330 (72.28)	1289 (74.60)	1490 (76.37)	< 0.001
Cotinine, ng/mL						< 0.001
> 10	2092 (26.10)	406 (20.28)	493 (24.61)	549 (29.09)	644 (30.32)
LOD-10	4309 (53.76)	1128 (56.34)	1079 (53.87)	960 (50.87)	1142 (53.77)
< LOD	1615 (20.15)	468 (23.38)	431 (21.52)	378 (20.03)	338 (15.91)
BMI, kg/m^2^						< 0.001
< 25	2361 (29.85)	673 (34.27)	593 (29.83)	556 (29.88)	539 (25.70)
25-29.9	2713 (34.30)	633 (32.23)	698 (35.11)	632 (33.96)	750 (35.77)
≥ 30	2836 (35.85)	658 (33.50)	697 (35.06)	673 (36.16)	808 (38.53)
Liver impairment	276 (3.45)	67 (3.35)	76 (3.80)	61 (3.24)	72 (3.40)	0.787
Renal impairment	3376 (42.13)	1131 (56.44)	883 (44.11)	682 (36.18)	680 (32.05)	< 0.001
Cancer	705 (8.81)	239 (11.94)	202 (10.10)	152 (8.06)	112 (5.28)	< 0.001
Diabetic	1475 (18.40)	525 (26.20)	356 (17.77)	299 (15.85)	295 (13.89)	< 0.001
Hypertension	3253 (40.58)	1016 (50.72)	798 (39.84)	682 (36.14)	757 (35.64)	< 0.001
Hypercholesterolemia	3086 (38.49)	838 (41.84)	777 (38.79)	675 (35.77)	796 (37.48)	< 0.001
CVD	789 (9.84)	334 (16.67)	175 (8.74)	141 (7.47)	139 (6.54)	< 0.001
CHF	219 (2.74)	114 (5.72)	45 (2.25)	32 (1.70)	28 (1.32)	< 0.001
CHD	289 (3.62)	128 (6.42)	69 (3.45)	54 (2.87)	38 (1.80)	< 0.001
Angina	178 (2.23)	69 (3.46)	46 (2.30)	38 (2.02)	25 (1.18)	< 0.001
MI	316 (3.95)	126 (6.30)	69 (3.45)	68 (3.61)	53 (2.50)	< 0.001
Stroke	278 (3.47)	123 (6.15)	64 (3.20)	44 (2.34)	47 (2.22)	< 0.001
Family history of CVD	991 (12.71)	274 (14.16)	240 (12.33)	207 (11.24)	270 (13.01)	0.053

FT3, free triiodothyronine; FT4, free thyroxine; TSH, thyroid-stimulating hormone; GED, general education development; LOD, limit of detection; BMI, body mass index; CVD, cardiovascular diseases; CHF, congestive heart failure; CHD, coronary heart disease; MI, myocardial infarction.

### Thyroid hormone levels and all-cause mortality

**Figure 2:**
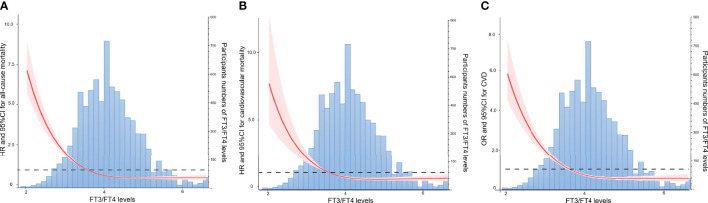
Association of the FT3/FT4 Ratio with All-cause Mortality, Cardiovascular Mortality, and CVD Risk by Unadjusted Restricted Cubic Splines. **(A)** Association of the FT3/FT4 Ratio with All-cause Mortality. **(B)** Association of the FT3/FT4 Ratio with Cardiovascular Mortality. **(C)** Association of the FT3/FT4 Ratio with CVD Risk.FT3, free triiodothyronine; FT4, free thyroxine.

The relationships between thyroid hormone levels and all-cause mortality are visually displayed using unadjusted restricted cubic splines. As shown in **Figure 2A** and **Supplementary Figures 1A, D**, the risk of all-cause mortality increased with increasing serum FT4 in a J-shaped pattern; in contrast, the risk of all-cause mortality dropped as serum FT3 and FT3/FT4 ratio increased in a reversed J-shaped pattern. After adjusting for all the covariates, the results of the multivariable Cox proportional hazards model were consistent with the unadjusted models, the risk of all-cause mortality increased by 15% (HR, 1.15; 95% CI, 1.09-1.22) for each SD increase in FT4, while the risk of all-cause mortality decreased by 19% (HR 0.81; 95% CI, 0.70-0.93) for each SD increase in FT3 and 23% (HR 0.77; 95% CI, 0.69-0.85) for each SD increase in the FT3/FT4 ratio (**Table 2**).

**Table 2:** Hazard Ratios of All-Cause and Cardiovascular Mortality by FT3, FT4 or FT3/FT4 Value Among Participants in NHANES III (2007-2012).

	FT3		FT4		FT3/FT4	
	HR (95% CI)	*P* value	HR (95% CI)	*P* value	HR (95% CI)	*P* value
All-cause mortality						
Unadjusted	0.40 (0.35-0.45)	< 0.001	1.12 (1.08-1.16)	< 0.001	0.51 (0.47-0.56)	< 0.001
Model 1	0.74 (0.65-0.84)	< 0.001	1.17 (1.11-1.23)	< 0.001	0.73 (0.67-0.81)	< 0.001
Model 2	0.76 (0.66-0.87)	< 0.001	1.17 (1.11-1.24)	< 0.001	0.73 (0.66-0.81)	< 0.001
Model 3	0.81 (0.70-0.93)	0.003	1.15 (1.09-1.22)	< 0.001	0.77 (0.69-0.85)	< 0.001
Cardiovascular mortality						
Unadjusted	0.37 (0.28-0.51)	< 0.001	1.13 (1.04-1.22)	0.005	0.52 (0.42-0.66)	< 0.001
Model 1	0.72 (0.52-1.00)	0.047	1.20 (1.05-1.37)	0.008	0.77 (0.61-0.96)	0.022
Model 2	0.71 (0.51-1.00)	0.047	1.20 (1.05-1.38)	0.008	0.75 (0.59-0.95)	0.015
Model 3	0.75 (0.53-1.06)	0.105	1.18 (1.01-1.39)	0.042	0.79 (0.62-1.00)	0.048

FT3, free triiodothyronine; FT4, free thyroxine.

Model 1: adjusted for age (6 groups) and sex (male or female).

Model 2: further adjusted for race/ethnicity (Mexican American, Non-Hispanic white, Non-Hispanic black or Other), education (< high school, high school/GED or > high school), marital status (married/living with partner, divorced/widowed/separated, or never married), serum cotinine (> 10, LOD-10 or < LOD ng/mL), alcohol drinking (yes or no).

Model 3: further adjusted for BMI (< 25, 25-29.9 and ≥ 30 kg/m^2^), hypertension (yes or no), diabetes (yes or no), hypercholesterolemia (yes or no), renal impairment (yes or no), history of CVD (yes or no), history of cancer (yes or no), history of liver impairment (yes or no), TSH (continuous), TPOAb (continuous), TgAb (continuous).

### Thyroid hormone levels and cardiovascular mortality

The findings for cardiovascular mortality were similar to those for all-cause mortality. As shown in **Figure 2B** and **Supplementary Figures 1B, E**, the risk of cardiovascular mortality increased with increasing serum FT4 in a J-shaped pattern; in contrast, the risk of cardiovascular mortality dropped as serum FT3 and FT3/FT4 ratio increased in a reversed J-shaped pattern. After adjusting for covariates, each SD increase in FT4 increased the risk of cardiovascular mortality by 18% (HR, 1.18; 95% CI, 1.01-1.39); in contrast, each SD increase in the FT3/FT4 ratio decreased the risk of cardiovascular mortality by 21% (HR, 0.79; 95% CI, 0.62-1.00). However, after adjustments were made to the full model (model 3), the relationship between FT3 and cardiovascular mortality was not statistically significant. This finding seemed to indicate that compared with FT3 or FT4 alone, the FT3/FT4 ratio may be a more precise and reasonable indicator of thyroid hormone metabolic variance and is related to cardiovascular mortality in the general population.

### Relationship between thyroid hormone levels and CVD and CVD subtypes

The relationships between thyroid hormone levels and total CVD are visually displayed using unadjusted restricted cubic splines. As shown in **Figure 2C** and **Supplementary Figures 1C, F**, the risk of CVD increased with increasing serum FT4 in a J-shaped pattern; in contrast, the risk of CVD dropped as serum FT3 and FT3/FT4 ratio increased in a reversed J-shaped pattern. As shown in **Table 3**, the fully adjusted model and unadjusted results were similar, with each 1 SD increment in FT4 increasing CVD risk by 17% (HR, 1.17; 95% CI, 1.08-1.27); in contrast, each 1 SD increment in the FT3/FT4 ratio decreased CVD risk by 18% (HR, 0.82; 95% CI, 0.74-0.92). After adjusting the full model (model 3), the relationship between FT3 and CVD risk was not statistically significant.

**Table 3:** Odds ratios of total and individual CVD risk by FT3, FT4 or FT3/FT4 levels among participants in NHANES III (2007-2012).

	FT3		FT4		FT3/FT4	
	OR (95% CI)	*P* value	OR (95% CI)	*P* value	OR (95% CI)	*P* value
CVD						
Unadjusted	0.48 (0.42-0.54)	< 0.001	1.12 (1.08-1.16)	< 0.001	0.57 (0.52-0.63)	< 0.001
Model 1	0.85 (0.74-0.97)	0.014	1.17 (1.11-1.23)	< 0.001	0.78 (0.71-0.86)	< 0.001
Model 2	0.86 (0.75-1.00)	0.042	1.17 (1.10-1.26)	< 0.001	0.79 (0.72-0.88)	< 0.001
Model 3	0.89 (0.76-1.03)	0.124	1.17 (1.08-1.27)	< 0.001	0.82 (0.74-0.92)	0.001
Congestive heart failure						
Unadjusted	0.34 (0.27-0.43)	< 0.001	1.17 (1.09-1.27)	< 0.001	0.43 (0.36-0.51)	< 0.001
Model 1	0.54 (0.42-0.69)	< 0.001	1.22 (1.12-1.33)	< 0.001	0.58 (0.48-0.69)	< 0.001
Model 2	0.54 (0.41-0.70)	< 0.001	1.21 (1.10-1.32)	< 0.001	0.58 (0.48-0.70)	< 0.001
Model 3	0.57 (0.43-0.77)	< 0.001	1.22 (1.08-1.37)	0.001	0.63 (0.51-0.77)	< 0.001
Coronary heart disease						
Unadjusted	0.38 (0.31-0.46)	< 0.001	1.14 (1.05-1.22)	0.001	0.49 (0.42-0.57)	< 0.001
Model 1	0.61 (0.49-0.76)	< 0.001	1.17 (1.06-1.29)	0.002	0.67 (0.57-0.78)	< 0.001
Model 2	0.60 (0.48-0.76)	< 0.001	1.17 (1.05-1.31)	0.005	0.67 (0.57-0.79)	< 0.001
Model 3	0.69 (0.54-0.89)	0.004	1.20 (1.06-1.37)	0.005	0.68 (0.57-0.82)	< 0.001
Angina pectoris						
Unadjusted	0.43 (0.34-0.55)	< 0.001	1.08 (0.97-1.20)	0.150	0.60 (0.50-0.72)	< 0.001
Model 1	0.70 (0.53-0.91)	0.009	1.07 (0.93-1.24)	0.334	0.79 (0.66-0.95)	0.013
Model 2	0.67 (0.51-0.89)	0.006	1.08 (0.92-1.26)	0.356	0.78 (0.64-0.95)	0.013
Model 3	0.73 (0.54-0.99)	0.042	1.04 (0.88-1.23)	0.656	0.83 (0.67-1.02)	0.070
Myocardial infarction						
Unadjusted	0.51 (0.42-0.61)	< 0.001	1.10 (1.02-1.19)	0.015	0.63 (0.55-0.73)	< 0.001
Model 1	0.79 (0.65-0.97)	0.024	1.11 (1.00-1.23)	0.045	0.82 (0.72-0.94)	0.005
Model 2	0.76 (0.62-0.95)	0.013	1.12 (1.01-1.25)	0.036	0.81 (0.70-0.94)	0.004
Model 3	0.84 (0.67-1.05)	0.132	1.13 (0.99-1.27)	0.062	0.83 (0.71-0.98)	0.025
Stroke						
Unadjusted	0.53 (0.44-0.64)	< 0.001	1.18 (1.09-1.27)	< 0.001	0.55 (0.47-0.64)	< 0.001
Model 1	0.97 (0.80-1.18)	0.744	1.20 (1.11-1.30)	< 0.001	0.77 (0.66-0.90)	0.001
Model 2	1.03 (0.87-1.23)	0.698	1.19 (1.10-1.30)	< 0.001	0.80 (0.68-0.94)	0.005
Model 3	1.05 (0.87-1.27)	0.622	1.20 (1.08-1.33)	0.001	0.83 (0.70-0.99)	0.036

FT3, free triiodothyronine; FT4, free thyroxine; CVD, cardiovascular diseases.

Model 1: adjusted for age (6 groups) and sex (male or female).

Model 2: further adjusted for race/ethnicity (Mexican American, non-Hispanic white, non-Hispanic black or other), education (< high school, high school/GED or > high school), marital status (married/living with partner, divorced/widowed/separated, or never married), serum cotinine (> 10, LOD-10 or < LOD ng/mL), alcohol drinking (yes or no).

Model 3: further adjusted for BMI (< 25, 25-29.9 and ≥ 30 kg/m^2^), hypertension (yes or no), diabetes (yes or no), hypercholesterolemia (yes or no), renal impairment (yes or no), family history of CVD (yes or no), history of cancer (yes or no), and history of liver impairment (yes or no), TSH (continuous), TPOAb (continuous), TgAb (continuous).

There are five common subtypes of CVD, including CHF, CHD, angina pectoris, MI, and stroke. After adjusting for all the covariates in logistic regression, we found that FT4 level was positively associated with the risk of CHF, CHD, and stroke, and the corresponding ORs and 95% CIs of each SD increment in FT4 were 1.22 (1.08-1.37), 1.20 (1.06-1.37), and 1.20 (1.08-1.33), respectively. In contrast, FT3 level was negatively associated with the risk of CHF, CHD, and angina pectoris, the corresponding ORs and 95% CIs of each SD increment in FT3 were 0.57 (0.43-0.77), 0.69 (0.54-0.89), and 0.73 (0.54-0.99); the FT3/FT4 ratio was negatively associated with the risk of CHF, CHD, MI, and stroke, the corresponding ORs and 95% CIs of each SD increment in the FT3/FT4 ratio were 0.63 (0.51-0.77), 0.68 (0.57-0.82), 0.83 (0.71-0.98), and 0.83 (0.70-0.99), respectively.

These findings seem to indicate that the correlation between the FT3/FT4 ratio and CVD is stronger than that of FT3 or FT4 only.

### FT3/FT4 ratio and stratified analyses

**Figure 3:**
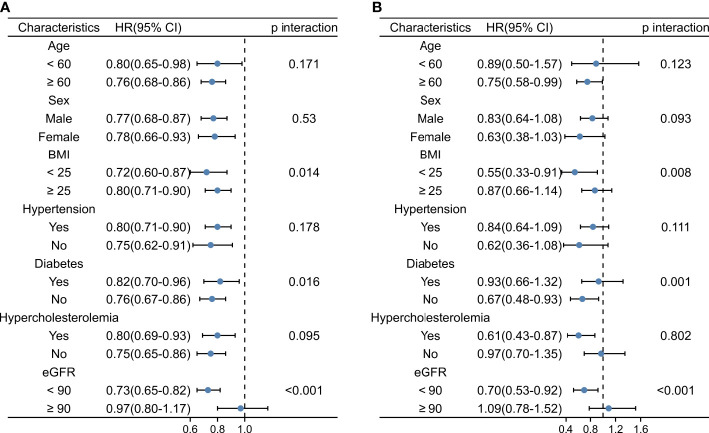
Stratified Analysis of the FT3/FT4 Ratio and Risk of All-cause Mortality and Cardiovascular Mortality. **(A)** Stratified Analysis of the FT3/FT4 Ratio and Risk of All-cause Mortality. **(B)** Stratified Analysis of the FT3/FT4 Ratio and Risk of Cardiovascular Mortality. FT3, free triiodothyronine; FT4, free thyroxine; BMI, body mass index; CVD, cardiovascular disease.

Stratified analysis of the FT3/FT4 ratio and the risks of mortality and total CVD is shown in **Figure 3** and **Supplementary Figure 2**. The stratified results were generally consistent with the overall result. A higher FT3/FT4 ratio was associated with a lower risk of all-cause mortality, cardiovascular mortality, and CVD risk across subgroups. There was no significant interaction between the FT3/FT4 ratio and sex, age, hypertension, or hyperlipidemia, but there was a significant interaction between the FT3/FT4 ratio and diabetes, eGFR, and BMI. The impact of the FT3/FT4 ratio on all-cause and cardiovascular mortality was stronger in the nondiabetic, renal impairment and BMI < 25 kg/m^2^ subgroups. Similarly, the link between the FT3/FT4 ratio and cardiovascular disease was stronger in the nondiabetic and renal impairment subgroups.

## Discussion

In this nationwide survey, we found a significant link between thyroid hormone levels and death and CVD risk. High FT4 levels increased the risk of all-cause mortality, cardiovascular mortality, and CVD, while high FT3 or a high FT3/FT4 ratio reduced the risk of all-cause mortality, cardiovascular mortality, and CVD.

Thyroid hormones mainly include T3 and T4. Thyroid hormone receptors are present in most tissues, as are thyroid hormone receptors in the heart and blood vessels. Thyroid hormones have a series of effects on the cardiovascular system. The underlying mechanisms are endothelial dysfunction, blood pressure changes, myocardial dysfunction, and dyslipidemia (1). Patients with severe hypothyroidism or hyperthyroidism are known to have an accelerated onset of CVD (1). Recent studies have linked changes in thyroid hormones within the reference range with adverse clinical outcomes in the general population. Cappola AR et al. enrolled 2,843 elderly community residents in the U.S. and followed them up for 18 years. They found that higher FT4 within the reference range was linked to an increased risk of all-cause mortality (24). Neves et al. included 7,116 adult subjects in the NHANES database and reported that lower serum FT3 within the normal thyroid function range was associated with an increased risk of cardiovascular death (28). Our findings support this notion, as we shown that, after adjusting for all variables, higher FT4 levels increased the risk of all-cause and cardiovascular mortality, higher FT3 levels decreased the risk of all-cause mortality, and FT3 had a trend toward being associated with lower cardiovascular mortality, but this was not statistically significant. In contrast with our study, Groothof D et al. included 6,054 community subjects and showed that higher FT3, not lower FT3, raised the risk of all-cause death in women (29). Ceresini G et al. included 815 community residents aged ≥ 65 who were followed up for 9 years and showed that neither FT3 nor FT4 was correlated with all-cause mortality (30). The contradictory findings might be because FT3 or FT4 alone does not represent global alterations in thyroid hormone metabolism (18).

Although T4 is the most abundant thyroid hormone in the circulation, the main thyroid hormone action comes from T3, and most circulating T3 is converted from peripheral T4 by deiodinase. Hence, the transformation of T4 to T3 is critical for the creation of circulating T3 and for the thyroid hormone effects on the cardiovascular system, a process that can be quantified in the FT3/FT4 ratio (31–33). The FT3/FT4 ratio may be a more precise and feasible indicator of thyroid hormone metabolic variability that is more strongly associated with prognosis than FT3 or FT4 alone (18). Pasqualetti G et al. showed that a reduced FT3/FT4 ratio was a valid prognostic parameter for higher mortality in hospitalized elderly patients (34). The FT3/FT4 ratio may be a useful indicator of higher mortality risk in patients diagnosed with DCM and is associated with worsening cardiac function (17). Similarly, in euthyroid individuals undergoing percutaneous coronary intervention with a prior cardiovascular event, the FT3/FT4 ratio could be a significant predictor of all-cause death and cardiac death (35). A low FT3/FT4 ratio is correlated with an increased risk of long-term cardiac death and major adverse cardiovascular and cerebrovascular events in euthyroid individuals with three-vessel coronary artery disease (18). However, the connection between the baseline FT3/FT4 ratio with CVD and whether it predicts long-term mortality is unclear. By integrating and analyzing the data from the large NHANES database, we propose for the first time that among U.S. adults, the FT3/FT4 ratio is associated with CVD and is an independent predictor of cardiovascular death and all-cause death. Further stratified analysis showed that the above results had good predictive ability in each subgroup.

After adjusting for all variables, higher FT4 was independently correlated with an elevated risk of CVD, while a higher FT3/FT4 ratio was independently correlated with a decreased risk of CVD, with a nonsignificant trend toward a lower CVD risk for FT3. Similar to our study, a large population-based survey showed that in the euthyroid general population, the risk of CVD during follow-up in the highest tertile of FT4 was 1.32 times higher than that in the lowest tertile (36). A correlation of FT3 with CVD in the general population has not been reported, but seemingly inconsistent with our study, Kim HJ et al. showed that high baseline FT3 levels in the general population were associated with the development of metabolic syndrome (37). Interestingly, after the development of CVD, the normal negative feedback regulation of thyroid function may be disrupted by the disease, resulting in decreased serum FT3 but no significant change in FT4 or TSH, this phenomenon is also known as the low-T3 syndrome (38–42). In these diseases, the reduction in T3 is likely due to reduced deiodination of the conversion of T4 to T3, changing T4 metabolism to greater amounts of physiologically inactive reverse T3 (43). Based on the above, we boldly speculate that in our study, high levels of FT4 may lead to the occurrence of CVD, and CVD causes a decrease in deiodinase activity, which in turn leads to a decrease in T3, so FT4 and CVD are positively correlated, while FT3 and the FT3/FT4 ratio are negatively correlated with CVD, but this needs to be confirmed by further prospective studies.

There are certain limitations to our research. First, this evaluation of the links between thyroid hormone levels and CVD was based on cross-sectional data and therefore could not establish a causal relationship. At the same time, we were unable to obtain data on unrecognized CVD in patients, which may affect the accuracy of the results. Still, due to our large sample size and good adjustment for collected confounders, we provide the best available evidence for assessing the associations between thyroid hormone levels and CVD in humans. Second, we cannot completely rule out measurement error, since a single serum thyroid assay was done on each volunteer, though thyroid hormone levels in a given person stay within a narrow range over time (44). Finally, although we controlled for many possible confounders, our findings may be affected by residual confounders, random error, or factors that were not controlled for, since the many factors that affect CVD and survival can bias the outcomes.

## Conclusions

In U.S. adults without prior thyroid disease, FT3, FT4, and the FT3/FT4 ratio were all independent predictors of all-cause death. FT4 and the FT3/FT4 ratio, but not FT3, were shown to be independent predictors of cardiovascular mortality and CVD risk. Along with the FT3 and FT4 concentrations, we should pay equal attention to the FT3/FT4 ratio in the general population.

## Data availability statement

Publicly available datasets were analyzed in this study. This data can be found here: www.cdc.gov/nchs/nhanes/index.htm/.

## Ethics statement

The studies involving human participants were reviewed and approved by the National Center for Health Statistics Ethics. The patients/participants provided their written informed consent to participate in this study. Written informed consent was obtained from the individual(s) for the publication of any potentially identifiable images or data included in this article.

## Author contributions

Conceptualization, XL and YL. Methodology, XL and YL. Software, XL, YL and NW. Data curation, XL, YL, YHZ and NW. Visualization, XL, DZ and YHZ. Validation, XL and YL. Writing the original draft, XL, YL and DZ. Writing, review, and editing, XL and YZ. Supervision, XL and YZ. Funding acquisition, YZ and YL. All authors agreed to the published version of this manuscript.

## Funding

This research was funded by the National Natural Science Foundation of China (grant 81770255 to YZ and grant 82000381 to YL) and the Open Project of Key Laboratory of Myocardial Ischemia, Ministry of Education (grant KF202103 to YZ and grant KF202216 to XL).

## Acknowledgments

We thank all authors for their contributions and support.

## Conflict of interest

The authors declare that the research was conducted in the absence of any commercial or financial relationships that could be construed as a potential conflict of interest.

## Publisher’s note

All claims expressed in this article are solely those of the authors and do not necessarily represent those of their affiliated organizations, or those of the publisher, the editors and the reviewers. Any product that may be evaluated in this article, or claim that may be made by its manufacturer, is not guaranteed or endorsed by the publisher.
