# Supplementary material for: FT3/FT4 ratio is correlated with all-cause mortality, cardiovascular mortality, and cardiovascular disease risk: NHANES 2007-2012
Source: Front Endocrinol (Lausanne). 2022 Aug 18;13:964822. doi: 10.3389/fendo.2022.964822 (PMC9433660; doi:10.3389/fendo.2022.964822)
Supplement: Supplementary file 1 [file DataSheet_1.docx]

**Supplementary Material**

Xueyan Lang^1, 2†^, Yilan Li^1, 2†^, Dandan Zhang^1, 2^, Yuheng Zhang^1^, Nilian Wu^1^, Yao Zhang^1, 2*^

**Supplementary Figure 1.** Association of the FT3 and FT4 Levels with All-cause Mortality, Cardiovascular Mortality, and CVD Risk by Unadjusted Restricted Cubic Splines.

**
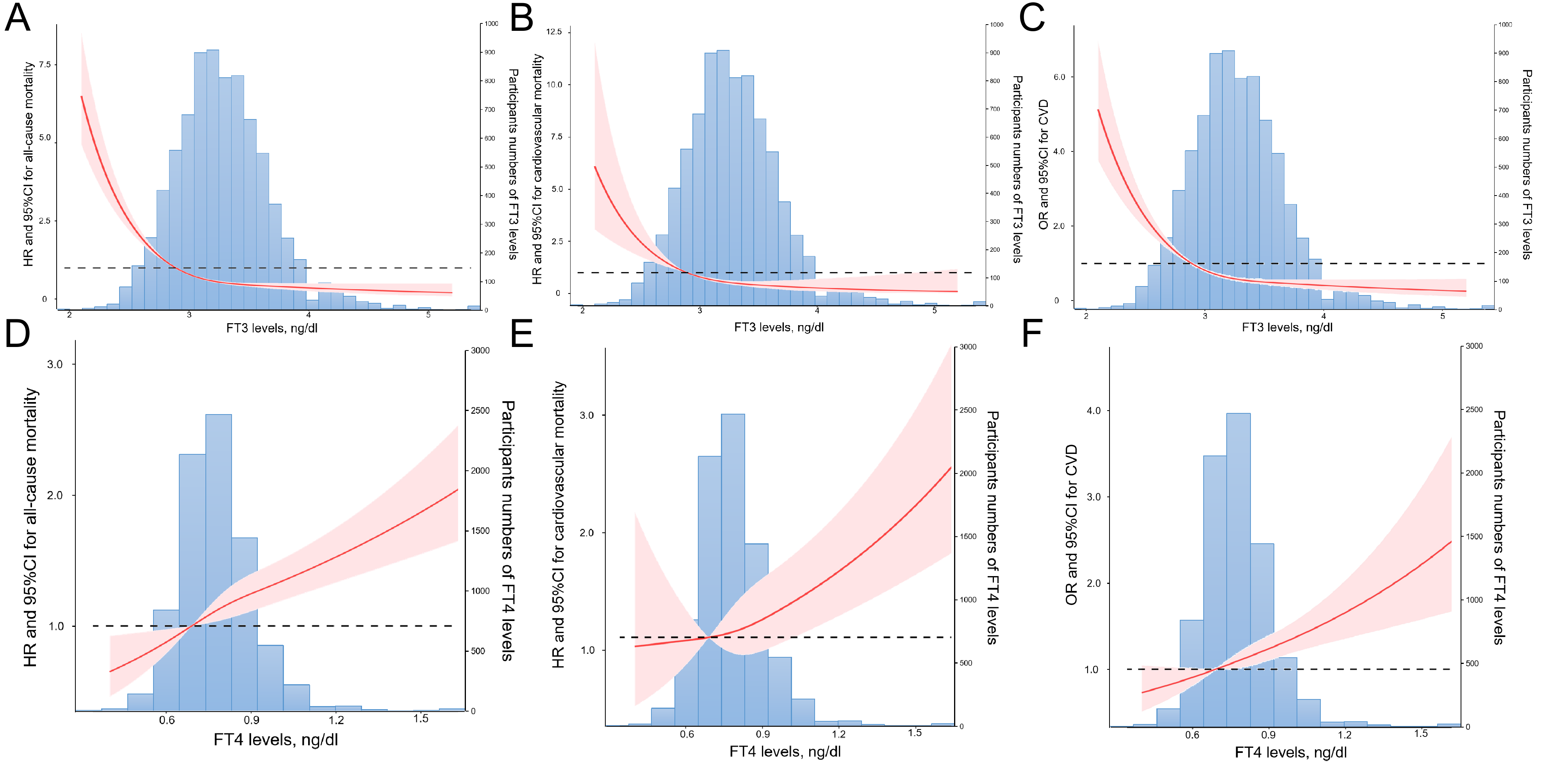
**

**Supplementary Figure 1.** Association of the FT4 levels with all-cause mortality (A) and cardiovascular mortality (B) and CVD risk (C). Association of the FT3 levels with all-cause mortality (D) and cardiovascular mortality (E) and CVD risk (F).

FT3, free triiodothyronine; FT4, free thyroxine.

**Supplementary Figure 2.** Stratified Analysis of the FT3/FT4 Ratio and CVD in NHANES Participants From 2007 to 2012.


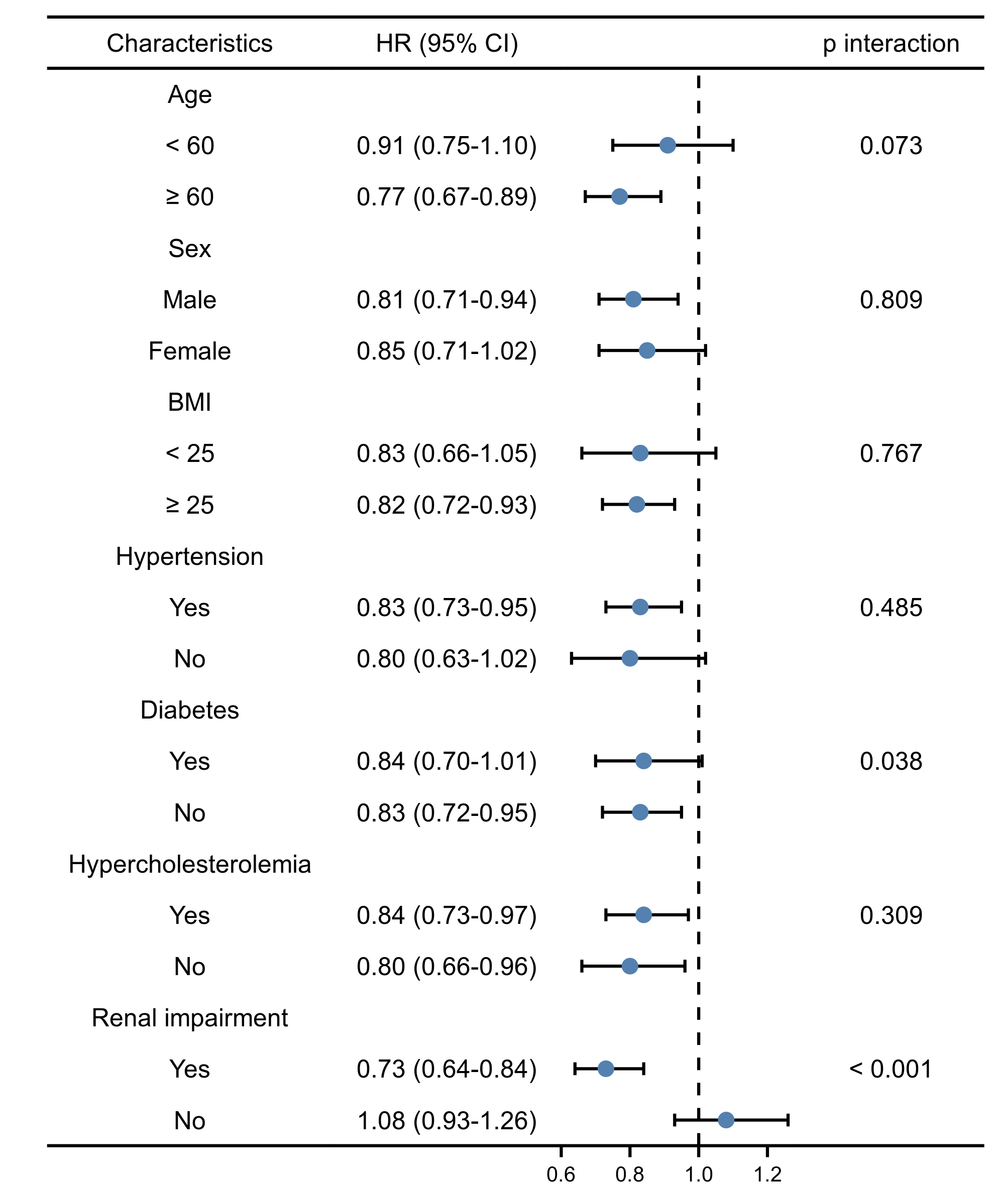


FT3, free triiodothyronine; FT4, free thyroxine; BMI, body mass index; CVD, cardiovascular disease
